# Thoracocervicofacial Emphysema after Heimlich's Maneuvre

**DOI:** 10.1155/2015/427320

**Published:** 2015-03-05

**Authors:** Salim Bouayed, Kishore Sandu, Pedro S. Teiga, Bassel Hallak

**Affiliations:** Department of Otorhinolaryngology, Head and Neck Surgery, Hospital of Sion, 1950 Sion, Switzerland

## Abstract

We report an extremely rare example of a thoracocervicofacial subcutaneous emphysema after Heimlich maneuver case.

## 1. Introduction

In 1974, Henry Heimlich described his life saving manoeuvre of abdominal infradiaphragmatic pressure to dislodge aspirated food from upper airways. The manoeuver of Heimlich consists in creating an increased intrathoracic pressure by means of an abrupt epigastric compression directed upwards [1], forcing expiry of the residual trapped intrapulmonary air followed by an expulsion of the foreign body in the airway. This forced air expiration, sometimes against a closed glottis, can be associated with complications which can be multiplied by the actual foreign body impaction in the upper aerodigestive tract. Here we report a case of subcutaneous thoracocervicofacial emphysema after Heimlich's maneuver.

## 2. Clinical Case

**Figure 1:**
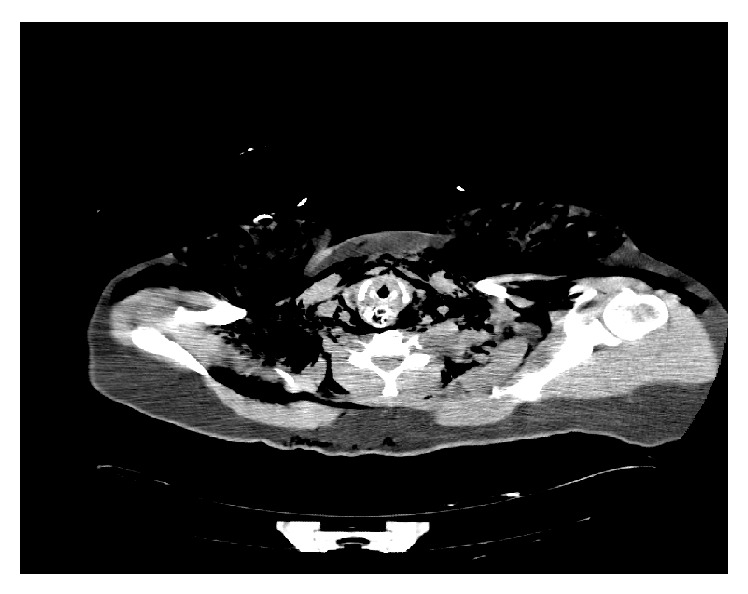
CT scan axial section showing the foreign body and subcutaneous emphysema.

**Figure 2:**
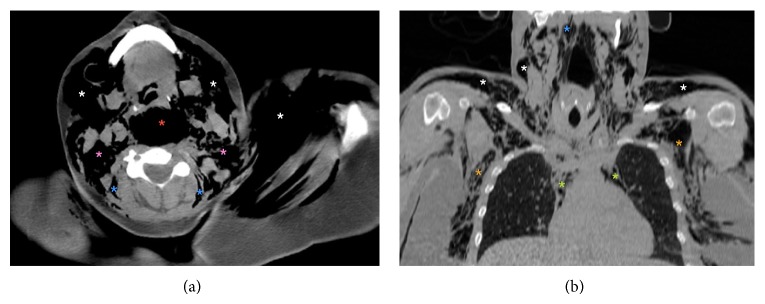
(a) Axial, (b)coronal cervicothoraco CT scan with 2D reconstruction obtained urgently.

**Figure 3:**
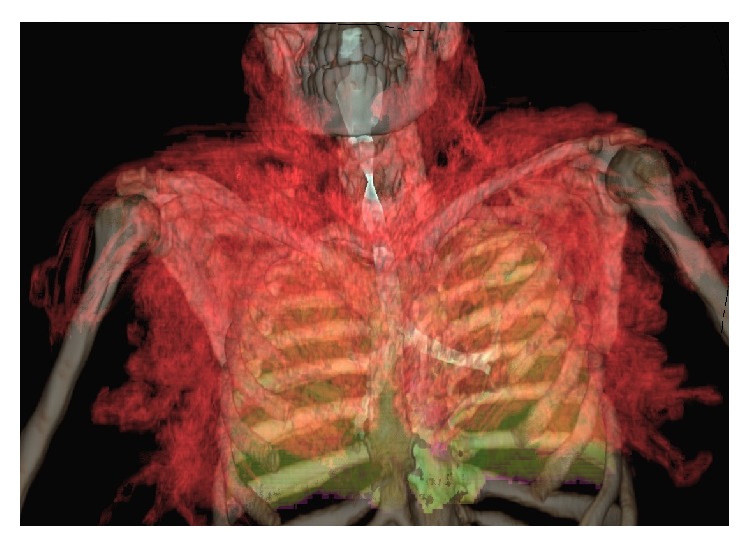
3D reconstruction from images of the cervical-thoracic CT obtained emergency shows the distribution of emphysema in relation to other structures aeric content. Emphysema is shown in red; the trachea and bronchi are shown in blue, light green lung.

A 45-year-old Caucasian woman, mentally disabled, living in an institution of special care, presented with an acute onset chocking with respiratory distress during her meal. The care-taker nurse had noticed that she had eaten a large piece of chicken meat. Instantaneously, the nurse performed Heimlich's maneuver on three separate occasions. Immediately after the maneuver, the acute respiratory distress partially resolved, though the patient developed subcutaneous emphysema extending from the thorax to the face closing the eyelids completely. The blood oxygen saturation was above adequate. A transnasal fibreoptic laryngoscopy showed salivary stasis in both piriform sinuses. There was no laryngeal edema and vocal cord mobility was conserved. An urgent cervicothoracic CT scan (Figure 1) done in the following hour revealed a three cm long foreign body of bone density located at the esophageal opening. In addition, massive subcutaneous emphysema was seen, cranially from the fat pad of Bichat extending posteriorly to the retropharyngeal space, the occipital region descending caudally to the axilla, and the mediastinum (Figures 2(a), 2(b), and 3). There the lung parenchyma was normal and there was no pleural effusion. A rigid pharyngoesophagoscopy was done 6 hours later extracting a large piece of bony chicken meat which was impacted in the right piriform sinus. On repeated endoscopy a 3 mm tear was seen at the apex of the right piriform sinus extending until the cricopharynx. Because the size of the tear was small we decided against an endoscopic repair of the tear only inserting a nasogastric feeding tube under endoscopic control. The patient was covered with amoxyclavulanic acid 1.2 g three times a day.

Twenty-four hours later, the patient redeveloped a progressive respiratory distress with increasing inflammatory parameters. The patient was febrile and had tachycardia (HR > 110/min). A new cervicothoracic CT scan was performed. It revealed a regression of the subcutaneous emphysema and the pneumomediastinum but showed evidence of bilateral pleural effusion and atelectasis. Bilateral intercostal drains were inserted in emergency. A new pharyngoesophagoscopy showed pus in the right piriform sinus. A right exploratory cervicotomy was performed to evacuate the abscess and showed no evidence of residual foreign body. The site was rinsed with dilute hydrogen peroxide and betadene^R^ and closed over 2 easy-flow drains. The wound was rinsed with dilute betadine solution 2 times a day for the next 3 days. Antibiotherapy (amoxicillin-clavulanate) was continued for 10 days. The thoracic tubes were pulled out at day 5. Over the next few days the inflammatory parameters settled and the general condition improved. The cervical drains were removed on the sixth day. A cervicothoracic CT scan performed on day 9 showed a complete resolution of the cervical pneumomediastinum, the pleural effusion, and the subcutaneous emphysema. A barium study done at 2 weeks was normal and the patient was restarted on feeds.

## 3. Discussion

Heimlich's maneuver is used commonly in case of foreign body blockage in the superior aerodigestive tract but has been associated with many complications reported in the medical literature.

Complications associated with this maneuver mentioned in the literature include vomiting, pharyngeal or oesophageal tears, and rib fractures. Other more serious complications described are esophagogastric and jejunal ruptures [2–4], thrombosis of the abdominal aorta [5], diaphragmatic hernia [6], and pneumomediastinum [4, 7]. The pneumomediastinum and the subcutaneous emphysema although rare, they can occur following bronchopleural and pharyngoesophageal tears [8].

Pharyngoesophageal perforations can be caused by sharp foreign body impactions, external trauma, caustic injuries, and iatrogenically induced endoscopic interventions. The impaction of pharyngeal or oesophageal foreign body is responsible for a perforation in 2% of the cases [9, 10]. To the best of our knowledge, Heimlich's maneuver performed for a sharp foreign body impaction leading to a secondary hypopharyngeal perforation has not yet been described in the literature. Following a foreign body impaction, subcutaneous emphysema on clinical examination and mediastinal emphysema on radiological imaging should evoke suspicion of a pharyngoesophageal tear. The emphysema can be exaggerated by raised intrathoracic and abdominal pressures during Heimlich's maneuver which is commonly advocated to relieve foreign body impactions in the upper aerodigestive tract. This is exactly what happened in our patient in whom Heimlich's maneuver unfortunately complicated a foreign body impaction causing a more serious pneumomediastinum. In our patient the pharyngeal perforation led to a fistula and subsequently mediastinitis. Prompt surgical drainage of the abscess, intercostal drains, and intravenous broad-spectrum antibiotics were given to treat the patient.

Subcutaneous emphysema usually regresses by itself over 3–10 days. Surgical exploration allows the release of emphysema, but it is important to drain this wound by an easy-flow, Penrose, or corrugated rubber drains. A tight closure without a drain will not allow the release of the air trapped within the subcutaneous planes. Oral feeds are started only when there is evidence of complete pharyngeal fistula healing on a barium swallow study and the inflammatory parameters settle. In case of pharyngeal or esophageal perforation, the traditional treatment is surgery. Many writers described the medical treatment without serious complications [10–12]. For Skinner et al., the treatment of perforation in the piriform sinus must be based on the extension [13]. High pharyngeal fistulas can be closed by an endoscopic approach, whereas distal or esophageal fistulas need open approach and closure. In our case, medical treatment failed probably because of extensive subcutaneous emphysema. It would have been ideal if we had extracted the sharp foreign body endoscopically and explored the neck during the same time to evacuate the emphysema which could have avoided the mediastinal complications.

## 4. Conclusions

Heimlich's maneuver is practiced commonly to relieve a trapped foreign body in the upper aerodigestive tract. However, its use in case of a sharp pointed foreign body may lead to an esophageal perforation with extensive cervicomediastinal emphysema, which warrants foreign body extraction and is combined with an open exploration.

## References

[1] Heimlich H. J. (1975). A life-saving maneuver to prevent food-choking. *The Journal of the American Medical Association*.

[2] Ayerdi J., Gupta S. K., Sampson L. N., Deshmukh N. (2002). Acute abdominal aortic thrombosis following the Heimlich maneuver. *Cardiovascular Surgery*.

[3] Gallardo A., Rosado R., Ramírez D., Medina P., Mezquita S., Sánchez J. (2003). Rupture of the lesser gastric curvature after a Heimlich maneuver. *Surgical endoscopy*.

[4] Meredith M. J., Liebowitz R. (1986). Rupture of the esophagus caused by the Heimlich maneuver. *Annals of Emergency Medicine*.

[5] Kirshner R. L., Green R. M. (1985). Acute thrombosis of abdominal aortic aneurysm subsequent to Heimlich maneuver: a case report. *Journal of Vascular Surgery*.

[6] Ujjin V., Ratanasit S., Nagendran T. (1984). Diaphragmatic hernia as a complication of the Heimlich maneuver. *International Surgery*.

[7] Rich G. H. (1980). Pneumomediastinum following the Heimlich maneuver. *Annals of Emergency Medicine*.

[8] Okada T., Sasaki F., Todo S. (2004). Perforation of the piriform recessus by a swallowed glass splinter presenting as pneumomediastinum in a child. *Pediatric Surgery International*.

[9] Nandi P., Ong G. B. (1978). Foreign body in the esophagus: review of 2394 cases. *British Journal of Surgery*.

[10] Radford P. J., Wells F. C. (1988). Perforation of the oesophagus by a swallowed foreign body presenting as a mediastinal and pulmonary mass. *Thorax*.

[11] Altorjay Á., Kiss J., Vörös A., Bohák Á. (1997). Nonoperative management of esophageal perforations: is it justified?. *Annals of Surgery*.

[12] Jones W. G., Ginsberg R. J. (1992). Esophageal perforation: a continuing challenge. *Annals of Thoracic Surgery*.

[13] Skinner D. B., Little A. G., DeMeester T. R. (1980). Management of esophageal perforation. *The American Journal of Surgery*.

